# The National Liver

**Published:** 1891-07-25

**Authors:** 


### The Hospital

A Weekly Journal of

**Science, Medicine, Nursing, and Philanthropy.**

*For Hospitals, Asylums, Infirmaries, Dispensaries, Medical Practitioners, Students, Nurses, and the Charitable Public.*

Vol. X.—No. 252.] [July 25, 1891.

### THE NATIONAL LIVER.

All the blood which circulates in the lower half of the human body, that is the half below the chest, is gathered up into the portal vein and by it discharged into the liver. The blood thus brought to the liver is venous blood, and is therefore loaded with a great variety of waste products from those organs of the body through which it has been circulating. It sometimes happens that the quantity of blood, with its accompanying waste matter, thus brought to the liver, is excessive. There is then produced a congestion of the liver, a sort of misery with which most town-bred people are quite familiar. Certain forms of disease so entirely upset the balance between the energy of the liver on the one hand, and the physiological demands made upon it on the other, that death is the inevitable result. All this is part of the daily experience of the physician; and one of his constant aims is to maintain a condition of just balance between the power of the liver and the work it is called upon to do for its owner.

Mr. Herbert Spencer has taught us to compare the nation with the individual; and a much less distinguished person has led us to look for "natural law in the spiritual world." We are, therefore, following excellent precedents when we are comparing physiological processes with political activities. The British nation may be said to have a liver; and we may consider that liver to be the poorer districts of its large cities and towns. That liver, a well known sociological physician, the Earl of Meath, tells us is now in a state of profound and dangerous congestion. To pursue the physiological metaphor, Lord Meath seems to think that the organ is growing portentously large, and, indeed, is threatening to entirely fill the abdominal cavity. If that be really so it is evident that the patient must be taken in hand at once, or death will certainly be the result.

Now assuming Lord Meath to have formed a sound diagnosis, it is very clear that the case is urgent and that a consultation is demanded; and we therefore invite all our properly qualified readers to assemble with us round the patient's bed. Our first business, it is obvious, is to carefully examine the sufferer and to find out whether or not Lord Meath's diagnosis is correct. Is the patient's liver really enlarged; and is the enlargement of such a degree that unless it is adequately reduced a prognosis of death must be given? These questions can only be answered by a careful consideration of the actual facts of the case.

Lord Meath calls attention to certain symptoms which he considers peculiarly urgent. He states that two members of the National Association for Promoting State Colonisation visited three of the docks of London on a particular morning not long ago. They there saw thousands of men standing about in a state of semi-starvation waiting for the chance of being employed. No sooner were the dock gates opened than those men, fierce as wild beasts, rushed to the opening, scrambling over one another's heads and trampling each other to the ground in their frantic efforts to get near the foreman, who was selecting the hands required for the day's work From those three docks alone 700 men were that morning sent away because there was no work for them to do. Probably very few of them had breakfasted, and perhaps hardly one in a score had in his pocket the price of a meal. Many of the 700 were husbands and fathers. It is stated that 8,000 men are daily turned away from the different London docks without employment; and it is probable that those 8,000, many of them having wives and children, represent at least 25,000 individuals. But the docks constitute only a small part of the labour market, even of London alone; and the labour market of London, huge though our city be, represents but a fraction of the whole market of British labour. If what has been described is not a symptom of acute and urgent congestion of the national liver, what is?

The Tower Hamlets—too familiar name—has a population of 450,000. Of these no fewer than 50,000 are described by Mr. Charles Booth as being in a chronic condition of "leisure, bounded very closely by the pressure of want." As many as 7,000 belong to the semi-criminal class, that is, persons who sometimes work and sometimes steal, according as fancy or necessity urges them. As many as 30,000 of the inhabitants of Tower Hamlets are described as "Irregular Poor"; they work when they can get work, and when they cannot get it they starve. As a sample of what is going on in the country, the Staffordshire and East Worcestershire nail makers are instanced by Lord Meath. So great is the pressure of poverty in those districts that "married women with large young families wield heavy hammers for twelve or fourteen hours a day. For a pittance of four or five shillings a week mothers abandon their duties and the instincts of affection, and do the work of slaves on a Cuban plantation. A man of sixty-eight, who has been a chain maker for fifty-four years, and earns five shillings and threepence a week, out of which he finds fire, shop, and tools, exclaimed to Mr. Burnett, 'I often feel inclined to put myself away.'" What a tough old hero he must have been not to have put himself away years before!

Another symptom of a most dangerous kind which Lord Meath points out is the incredibly low rate of wages in many industries. For making a hundred and forty four match-boxes and finding her own paste a girl is paid twopence farthing. The standard rate of pay for making paper bags is fivepence halfpenny a thousand. Sack makers are paid threepence a-dozen for their work, whilst the constructors of low-class shirts receive twopence each and find their own cotton. Every shirt takes more than two hours to make. A fancy box-maker, after giving six months work without wages, can earn from three shillings and sixpence to five shillings a week. But perhaps the maximum of absurdity and misery is reached by the maker of pill-boxes. The unfortunate creature who thus ministers to the profit of the doctor and the comfort of his patient puts together no fewer than 5,184 pill-boxes for a single shilling. What a miracle of work for a still greater miracle of pay! It is enough to make one abjure pills and pill-boxes for ever.

The foregoing are some of the leading symptoms from which the national patient is suffering. There cannot be much doubt or difficulty about the diagnosis. One of the most vital parts of the organism is acutely and dangerously congested. Do the national doctors and the many friends of the patient realise his condition? If they do, something may be done to avert the danger and bring about a cure. If they do not, there is but one ending to the business: the patient will die, just as other great nations have died, from internal breakdown and disorganisation. Is there any single doctor among our politicians and social reformers who has sufficient brains and energy to deal with the case? Both brains and energy of the very first order are imperatively demanded. Mere wise head shakings and pathetic lamentations are entirely out of place. The head-shakers and the pathetic lamenters must be got rid of; and if there be a practical State doctor to be found anywhere, no matter who he may be, he must be called in, and the patient entrusted to his care.

Lord Meath has a remedy: it is State colonisation. Let those who are interested in the great, venerable, and noble patient consider the nature of the remedy. It is not colonisation by individuals or associations, philanthropic or otherwise. In other words, it is not the administration of a pill by the doctor or of a bowl of slops by a grandmotherly nurse: what is insisted upon is a great, mighty, and successful effort which is to be made by the patient himself. He must get out of bed, leave his room, and trust to energetic exercise and fresh air to relieve him of his dangerous internal congestion. The remedy is distinctly rational and scientific. It promises a cure on natural lines; a cure which may, therefore, be both complete and permanent. Lord Meath shows us how easily the cure may be effected if the nation will but rouse itself to make the effort. We have millions of acres of land in our colonies waiting to be cultivated; we have millions of money in our larger national exchequer waiting to be employed. It is for the State to carry our superfluous workers to the work which is waiting to be done in our colonies, and there to nurse them a little until they can walk alone. Lord Meath's pamphlet on State colonisation discusses at length what we have here tried to deal with in the briefest possible way. Both the subject and the pamphlet are worthy of immediate and earnest consideration.

